# Attitudes towards schizophrenia and associated factors among community members in Hossana town: a mixed method study

**DOI:** 10.1186/s12888-023-04555-9

**Published:** 2023-01-28

**Authors:** Ayano Shanko, Lonsako Abute, Temesgen Tamirat

**Affiliations:** 1 Department of Internal Medicine, College of Medicine and Health Sciences, Wachemo University, Hossana, Ethiopia; 2 Public Health Department, College of Medicine and Health Sciences, Wachemo University, Hossana, Ethiopia

**Keywords:** Awareness, Attitude, Schizophrenia, Hossana, Southern Ethiopia

## Abstract

**Background:**

Mental health disorders have been identified as being one of the public health issues throughout the world. More than 24 million people worldwide suffer from schizophrenia. However, there is little information about the attitude toward people with Schizophrenia in Ethiopia.

**Objective:**

This study aimed to assess attitudes toward people with Schizophrenia and associated factors among residents of Hossana town, Southern Ethiopia.

**Methods:**

A community-based cross-sectional study was conducted with qualitative and quantitative data collection approaches among 417 households and three FGDs. The interviewer-administered standard tool was used to collect the data. Descriptive statistics like frequency, mean, and median are computed. A binary logistic regression model was used to identify factors affecting community perception and attitude toward people with schizophrenia.

**Results:**

Of the study participants, 194 (46.5%) had positive attitudes toward people with schizophrenia. Eccentric behavior and wandering were the most commonly mentioned manifestation. Besides, Substance misuse, loss of loved ones, and conflict with family as the perceived cause, and spiritual or traditional methods as the preferred treatment for people with schizophrenia. Moreover, participants with no family history of schizophrenia were six times [6.3(2.55–15.77)] more likely to develop a negative attitude towards schizophrenia than those with a family history of schizophrenia.

**Conclusion:**

In this study, the findings of this study indicate more than half of the participants had a negative attitude toward people with schizophrenia.

Eccentric behavior and wandering were the most commonly mentioned manifestation. Substance misuse, loss of loved ones, and conflict with family have been perceived causes of schizophrenia. Spiritual/traditional places were preferred places for the treatment. Having a family history of schizophrenia was the only factor associated with attitude towards schizophrenia. Therefore, due attention should be given to changing negative attitudes towards schizophrenia, reducing potential schizophrenia-predisposing factors, and enhancing community awareness to seek medical help as early as possible when such kinds of events occur.

## Introduction

Schizophrenia is a chronic, severe mental disorder that affects the way a person thinks, acts, expresses emotions, perceives reality, and relates to others [1]. Significant cognitive, perceptual, behavioral, and emotional abnormalities characterize this severe mental illness. Globally, just 1% of people are affected, making it the eighth most common reason for years spent with a disability [2, 3]. It is one of the most prevalent mental illnesses that has an impact on someone's capacity for learning and working [4, 5]. Schizophrenia affects approximately 24 million people or 1 in 300 people (0.32%) worldwide and the rate is 1 in 222 people (0.45%) among adults [6].

It is more prevalent in Africa, which has 0.5% of the world's population [7]. The general public has a wide variety of traditional beliefs about the causes of schizophrenia (including, among others, demon possession, bewitching, evil spirit, evil eye, God's will, sorcery, curse, and the imposition of just punishment) [8–10]. Schizophrenia patients have a two- to three-fold higher risk of dying young than the overall population [7]. This is because it exposes people to a variety of ailments, including cardiovascular, metabolic, infectious, and physical problems [11].

Many participants in a study performed in Pakistan held common views regarding topics like God's will (32.3%), superstitions (33.1%), loneliness (24.8%), and unemployment (19.3%), according to the study. [12]. Witchcraft/evil spirits and divine punishment were also supported as causes of schizophrenia by 94% and 66% of the community members, respectively, in addition to that finding from the Zaire community in Ghana [13]. Early detection, help-seeking behavior, adherence to treatment, and how people with schizophrenia integrate into society can all be impacted by the context of ideas and perceptions that patients, their families, and the community hold regarding the origins of schizophrenia [14, 15].

Only 35% of African patients with schizophrenia have received treatment from a cutting-edge mental health facility. An unfavorable perspective of the causes of mental illnesses and the types of therapies available exacerbates the terrible practices in locating appropriate therapy for schizophrenia. The severity of the treatment gap is demonstrated by the percentage of people with mental illness receiving ineffective care, which varies from 75% in South Africa to more than 90% in Nigeria [16]. One of the top 10 causes of morbidity in Ethiopia is schizophrenia, which is becoming more prevalent as a public health concern. Although schizophrenia has the aforementioned effects on public health, the issue has not gotten enough attention, especially in Ethiopia. Since it has long been believed that serious mental illness is caused by demon possession or the evil eye, this perception of mental illness in Ethiopia has been influenced [17]. In Ethiopia, where infectious diseases that can be prevented are highly common and receive a lot of attention, schizophrenia is still viewed as a non-life-threatening issue and as a result, is not given much attention. 12% of Ethiopians experience mental health issues in some way, with 2% reporting severe cases [18, 19].

The way people in the community view mental illness has an impact on whether or not individuals with schizophrenia seek the right kind of care. Inappropriate therapies are also influenced by a lack of information, a combination of traditional rites and modern intervention techniques, and other factors [20]. Therefore, this study aimed to assess the status of the community awareness and attitude towards people with schizophrenia among residents of Hossana town, Southern Ethiopia.

## Methods and materials

### Study area and period

This study was conducted in Hossana Town, Hadiya zone Southern Ethiopia from May 11^th^ to June 29, 2022. Hossana Town is located in the Hadiya zone in southern Ethiopia. It is 232 KM far from Addis Ababa, the Capital of Ethiopia. It has six kebeles; the lowest administrative divisions. The total population living in town is 117,362; males 57,574 and females 59,855.

### Study design

The community-based cross-sectional study design was used by employing both quantitative and qualitative data collection approaches.

### Population

All the households living in Hossana town were used as the source population. All the individuals from sampled households who lived for at least six months in the area, whose age ≥ 18 years, and available during data collection were included in the study population. But, those who were critically ill and unable to respond were excluded from the study.

### Sample size and sampling techniques

A single population proportion formula was used to determine the sample size for this study. The following assumption was used: the proportion of the community's negative attitude towards schizophrenia was 50%, 95% confidence level (1.96), 5% margin of error, and 10% non-response rate. As a result, the calculated sample size for this study was 422 individuals. Among six kebeles in Hosanna town, three kebeles were randomly selected for the quantitative part. The selected kebeeles were Sechduna, Melamba and Betel. Then the sample size was proportionally allocated to the three kebeles based on the size of the households in each kebeles. A systematic random sampling technique was used to select households based on the sampling frame prepared. K^th^ – interval was calculated to know the skipping interval and then the first household was selected by using the lottery method from a one-to-K^th^ interval. After knowing the first household, the next and other households were obtained by adding K^th^-interval. Finally, household heads/spouses or age ≥ years participants were interviewed by the data collectors.

Three focus group discussions (FGD) were conducted to explore factors related to attitude towards schizophrenia. One FGD participants were from community leaders, the second FGD participants were from religious leaders and the third FGD participant was from community members. For each of the FGD study participants were purposively selected. A homogenous group of 8–12 participants per group was established and it lasts 1–2 h per group discussion.

### Variables

Dependent variable: attitude towards schizophrenia.

Independent variables: socio-demographic factors, source of information.

### Operational definition

#### Schizophrenia

A serious mental disorder in which people interpret reality abnormally and that affects how a person thinks, feels, and behaves.

#### Attitude towards schizophrenia

It was measured based on 13 attitude-related questions. To categorize community attitudes towards people with schizophrenia, we used the demarcation threshold formula. i.e. To categorize community attitudes towards people with schizophrenia, we used the demarcation threshold formula.

1 $$\frac{total\;highest\;score-total\;lowest\;score\;}2{+\;total\;lowest\;score}$$

So, ≤ 34 scores were considered as having a positive attitude.

### Data collection tools and procedures

After a review of various kinds of literature, the tools were initially developed in English, then translated into Amharic, and finally back into English to ensure consistency. Before translating the questionnaire was tested for internal consistency (reliability) with Cronbach's Alpha test (0.7). And after translating the questionnaire, Cronbach’s Alpha test was 6.9. Interviewer- administered data collection method was used to collect data from the participants. The questionnaire was first-pretested in the Kebele that was not part of the study.

The questionnaire included socio-demographic factors, 13 attitude-related questions, and sources of information. The questionnaire with 13 items of five-point Likert scales (from 1 to 5): (1 = strongly agree, 2 = agree, 3 = neutral, 4 = disagree, and 5 = strongly disagree) was used to assess the community attitude toward people with schizophrenia. Five trained Bsc nurses as data collectors and two senior public health professionals as supervisors were involved in the study. To improve comprehension of the questionnaire and deal with difficulties, training was offered. It was also supposed to cover the participants' information's confidentiality and privacy.

For qualitative data collection; a safe, quiet and comfortable place was selected. FGD guide was used and discussions were apprehended to explore their information about schizophrenia and the main source of information, what participants perceive about the cause of schizophrenia, what the participants feel about the disease and its consequences, what about the transmission of the disease, common manifestation of schizophrenia, whether there is modern medication for schizophrenia and what the preferable place of treatment for the patients with schizophrenia and its relation with family history. A moderator manages the discussions by organizing the topic, controlling the dominants, allowing all group members to share his/her idea, and preventing side conversations and time management. There was a recorder that helps to generate transcription to identify speakers because many speakers and it is difficult to distinguish. There was also a note taker to describe participants, setting, physical, behavioral and facial expressions, and nonverbal interaction among participants.

### Data quality control

Two-day intensive training was given to the data collectors and supervisors. Before actual data collection, the tool was pre-tested on 5% of the actual sample size in Arada kebele. During and after data collection the questionnaire was checked for its completeness.

### Data process and analysis

After checking for the data completeness, the data was entered into Epi-Data version 3.1 software and then exported to SPSS version 26.0 software for further analysis. Descriptive statistics like percentages and proportions were calculated. Tables and texts were used to present the results. A binary logistic regression analysis was done. Bivariate analysis was conducted to identify variables candidate for multivariate analysis based on a p-value of < 0.25 cut-off. Multivariate analysis was done to identify variables significantly associated with the outcome variable at p-value < 0.05. Adjusted odds ratios (AOR) with a 95% confidence interval and a p-value of less than 0.05 were employed in the multivariable logistic regression to identify the factors significantly associated with attitude towards schizophrenia. Qualitative data from the FGDs were transcribed verbatim. The analyses involved structured coding procedures and thematic characterizations of the coded segments [21]. After reading through the text, the principal investigator analyzed the transcriptions using the coding reliability approach of thematic analysis [22]. Initial coding was performed by a principal investigator, using both inductive and deductive approaches [23]. After the initial coding was completed, the analysis included code categorization and it was analyzed with sub-them and used to triangulate quantitative data.

### Ethical consideration

Ethical approval committees of Wachemo University have approved this study according to the relevant guidelines and regulations of the university as indicated by approval number Ref. WCU/246/2022 and following the Declaration of Helsinki. After getting approval, informed consent was gained from each participant after it was made clear that there was no need to collect any other kind of data beyond verbal responses, such as blood or bodily fluids. They have been informed that we are free to end our participation at any moment and without penalty. Participants' privacy was also protected at all times during the research process.

## Result

### Socio-demographic characteristics

A total of 417 respondents participated in this study which yields a response rate of 98.8%. Of this 41% of them were in the age group of 20–30 and Hadiya and Protestant were the dominant ethnicity and religion in the study area. The majority of the participants were married and only 8.9% of the participants have a family history of mental illness (Table 1).

**Table 1:** Socio-demographic characteristics of respondents residing in Hosanna, Southern Ethiopia, 2022

Particular	Category response	Frequency	Percent (%)
Gender	Male	231	55.3
	Female	186	44.6%
Religion	Protestant	271	65
	Orthodox	125	30
	Muslim	12	3
	Catholic	9	2
Age groups	< 20	84	20
	20–30	174	41.7
	31–40	123	29.5
	> 40	36	8.6
Ethnicity	Hadiya	258	62
	Kambata	71	17
	Gurage	52	12.5
	Amahara	36	8.5
Marital status	Single	41	9.8
	Married	376	90.2
Education status	No formal education	89	21.4
	Primary(grade 1–8)	121	29
	Secondary (grade 9–12)	106	25.4
	College and above	101	24.2
Family history of mental illness	Yes	37	8.9
	No	380	91.9
Occupational status	Housewife	100	24
	Merchant	82	19.7
	Government employee	117	28
	Daily laborer	60	14.4
	Others	58	13.9
Monthly income(EBirr)	< 1000	62	15
	1000–2000	179	43
	> 2000	176	42

*EBirr* Ethiopian Birr

### Source of information about schizophrenia

Of the total participants, 83% had ever heard about schizophrenia. The main sources of information for the participants were mass media (40%) and their friends (33%). About 70% of them agreed that schizophrenia is treatable. Regarding causes of schizophrenia; 89.9% of substance misuse causes, 67.8% loss of loved ones, and 60.7% conflict with family cause were major ones respectively. As a preferable place of treatment, spiritual/ traditional 88.5% was the leading place for treatment of schizophrenia followed by health facilities 45%. Eccentric behavior 83% and wandering 71% were the major responses of the participants about the common manifestation of schizophrenia (Table 2). Here are qualitative findings that support the above findings:

**Table 2:** Source of information about schizophrenia among respondents residing in Hosanna, Southern Ethiopia, 2022

Variables	Categories	Frequency	Percentage (%)
Ever heard about schizophrenia	Yes	346	83
	No	71	17
The main source of information	Mass media	138	40
	Religious institutions	31	9
	Friends	114	33
	Family members	63	18
Is schizophrenia treatable?	Yes	292	70
	No	125	30
Schizophrenia as a mental health disorder	Yes	329	79
	No	88	21
Schizophrenia is the most serious mental disorder	Yes	371	89
	No	46	11
Known cause of schizophrenia	Yes	259	62
	No	158	38
Perceived causes schizophrenia	Head injury	212	50.8
	Physical illness	198	47.5
	Genetic cause	80	19.2
	Substance misuse cause	375	89.9
	The loss of a loved one causes	283	67.8
	Conflict with family cause	253	60.7
	Punishment by God cause	117	28
	Evil spirit cause illness	104	25
	Poverty cause illness	92	22
Preferred place of treatment	Family(psychological)	142	34
	Spiritual/ Traditional	369	88.5
	Biological	87	20.8
	Health facility	188	45
	Not treatable	46	11
A common manifestation of schizophrenia	Aggression/destructiveness	171	41
	Talkativeness	284	68
	Eccentric behavior	346	83
	Wandering	296	71
	Self-neglect	288	69
	Restlessness/anxiety	221	53
	Insomnia	196	47
	Loss of concision	153	36.7

FGD findings reveal that most communities believed that health institutions as preferable places for the treatment of schizophrenia and at the same time they also believe that spiritual treatment is an alternative. *“46 years aged male FGD participant,……..most of the patients with mental illness were better to take them to health institution for treatment but also I believe in spiritual treatment. Because I know one mental patient was going to the spiritual place and know he is better than from the previous condition".*

Most communities believe that mental health problems as the long-term consequence of substance misuse. Here below the qualitative FGD finding supports substance misuse as a cause of schizophrenia. *"38 years FGD female participant…. most of the time people with mental illness were previously chatted chewers, cigarette smokers, and other substance misuses. For example in my village one 23 aged boy passed his time with his friends who were chatting chewers, cigarette smokers, and even drinking excessive alcohol. Currently, a few of them stop that behavior and they are healthy but it comes to be a mental health problem. So for any mental health problem like* schizophrenia *substance misuse is the major cause".*

Also, there are community groups who believe that schizophrenia is exclusively the result of evil spirits or due to punishment by God causes. Also, there is a belief in the community as the mental problem may come due to bad things in the patient's background the family or family curse, and qualitative FGD finding is indicated below. *"43 years FGD female participant…… the cause for schizophrenia is an evil spirit. Different people may answer different answers but I believe that the cause of schizophrenia is evil spirits or punishment by God or it might be due to some bad things in his/her family background/ family curse”.*

### Attitude about schizophrenia

Of the total participants, 53.5% of them have a negative attitude toward schizophrenia. About 66.4% of the participants agree that patients with schizophrenia were dangerous and greater than half (54.4%) of the participants agree that individuals with schizophrenia are simply weak-willed, unmotivated people (Tabe: 3). This quantitative finding is supported by qualitative study FGD finding: there are FGD participants who believe that patients with schizophrenia were dangerous for healthy communities as “……0.34 years female ……if you ask me about their behavior, they are too dangerous for the other healthy community and even I fear them so much. When I cross the road and if I get them I turn to another direction due to fear them.” There is also another participant who believes that individuals with schizophrenia are simply weak-willed and apathetic people. They said the reason is characteristics of evil spirits “…0.40 years old male…….. majority of the patients with schizophrenia were bored, uninterested in everything, they don’t want to talk with everybody even with his/her parents. This is a common characteristic that individuals affected by demon/evil spirit shows”.

In this study, a majority (62.1%) of them disagree that genetics is the primary factor in the development of schizophrenia (Table 3). There is also a qualitative study explored by FGD that supports schizophrenia as not genetic but it might happen due to curse and punishment for crimes in the patient’s family. "………0.48 years male participants ……. I did not agree with idea that schizophrenia is a genetic disease since it comes due to the evil eye or God's will or magic. But it might sometimes happen if there is curse and punishment for crimes in the patient’s family”. Other FGD participants said that schizophrenia cannot happen or transmitted by family “……….. 39 years female participants…….it is not related to family history because I know one patient with schizophrenia in my village but still no one from that comes to develop schizophrenia.

**Table 3:** Attitude about schizophrenia among respondents residing in Hosanna, Southern Ethiopia, 2022

Items	Strongly agree	Agree	Neutral	Disagree	Strongly disagree
Do you agree as soon as a person shows signs of schizophrenia, he or she should be hospitalized?	60(14.4)	182(43.6)	56(13.4)	94(22.5)	25(6)
Do you agree schizophrenia is caused by substance abuse?	132(31.7)	236(56.6)	18(4.3)	31(7.4)	0(0)
Do you agree individuals with schizophrenia are simply weak-willed, unmotivated people?	69(16.5)	227(54.4)	43(10.3)	69(16.5)	9(2.2)
Do you agree if treated and medicated, people with schizophrenia can function fairly typically in society?	97(23.3)	182(43.6)	72(17.3)	57(13.7)	9(2.2)
Do you agree people with schizophrenia are dangerous	114(27.3)	277(66.4)	0(0)	26(6.2)	0(0)
Do you agree with a person with schizophrenia's social problems are their fault because they isolate themselves from others?	69(16.5)	185(44.4)	64(15.3)	90(21.6)	9(2.2)
Do you agree genetics are the primary factor in the development of schizophrenia?	18(4.3)	44(10.6)	65(15.6)	259(62.1)	31(7.4)
Do you agree most people with schizophrenia are poor?	9(2.2)	43(10.3)	17(4.1)	291(69.8)	57(13.7)
Do you agree individuals with schizophrenia do not need medication; they just need to change their thought processes and behaviors?	18(4.3)	35(8.4)	58(13.9)	223(53.5)	83(19.9)
Do you agree individuals with schizophrenia are victims of their disease and should be treated with sympathy?	70(16.8)	217(52)	44(10.6)	77(18.5)	9(2.2)
Do you agree people with schizophrenia have abnormal behavioral patterns?	109(26.1)	277(66.4)	15(3.6)	9(2.2)	7(1.7)
Do you agree people with schizophrenia should have the same educational, occupational, and social opportunities as “normal” individuals?	27(6.5)	44(10.6)	58(13.9)	237(56.8)	51(12.2)
Do you agree most people fear people with schizophrenia?	70(18)	95(24.4)	39(10)	161(41.4)	52(12.2)

Most (56%) of the participants disagree that people with schizophrenia should have the same educational, occupational, and social opportunities as "normal" individuals (Table 3). This finding is similar to the idea revealed through FDG from a qualitative study. “..……35 years male participants………I did not expect those patients with schizophrenia to come to be normal and have the same educational and occupational opportunities as normal individuals. Since they already deviate from normal conditions if they don't get mercy from GOD they have no chance to be normal and live day-to-day routine life as normal individuals. In other 43 years old male participants………..those patients with schizophrenia cannot work effectively as normal individuals because they are mentally handicapped due to punishment for their crimes or their families' evil acts.

Greater than half (53.5) of the participants agree that schizophrenia needs medication. This finding is consistent with the FGD result that schizophrenia is a treatable disease by modern medication in health institutions and also they reveal that those individuals with schizophrenia will be healthy as another person and can work and be productive. “29 years aged male…… most of the communities’ regard to attitude for schizophrenic is not good. But I know that it is treatable by proper health professionals in health institutions and those individuals with schizophrenia will be healthy as other people and can work and be productive. Most communities even stigmatize/isolate them and put them in another locked room singly. That is not advisable according to my belief".

Greater than half of the participants agree that individuals with schizophrenia are victims of their disease and should be treated with understanding. This response is in line with qualitative findings…….."35 years old female ………. For sure individuals, those with mental illness were victims due to the disease and the majority of the community has a negative attitude toward them. If you ask me this is not good! I fear approaching them because they may harm you if you did not know their behavior. That is why they are the victim in the community".

### Factors that affect Awareness and attitudes toward Schizophrenia

In bivariate regression analysis; sex, education, and family history of schizophrenia were identified as candidates for the multivariate binary logistic regression. During multivariate binary logistic regression, only a family history of schizophrenia remained significantly and independently associated with Awareness and attitudes toward Schizophrenia. Those communities with no family history of schizophrenia were six times [6.3(2.55–15.77)] more likely to develop a negative attitude towards schizophrenia than those with a family history of schizophrenia (Table 4).

**Table 4:** Factors that affect Awareness and attitudes toward Schizophrenia among respondents residing in Hosanna, Southern Ethiopia, 2022

Variables	Categories	Attitude towards schizophrenia,		COR(95%CI)	AOR(95%CI)	*p*-value
		Positive attitude, *n* = 194	Negative attitude, *n* = 223			
Age	< 20	39(20.1)	45(20.2)	1		
	20–30	81(41.8)	93(41.7)	0.99(0.59–1.67)		
	31–40	54(27.8)	69(30.9)	1.1(0.63–1.93)		
	> 40	20(10.3)	16(7.2)	0.7(0.31–1.52)		
Sex	Male	98(50.5)	133(59.6)	1	1	
	Female	96(49.5)	90(40.4)	0.69(0.47–1.02)	0.7(0.466–1.05)	0.08
Marital status	Single	24(12.4)	17(7.6)	1		
	Married	170(87.6)	206(92.4)	0.58(0.304–1.124)		
Education	No formal education	36(18.6)	53(23.8)	1	1	
	Primary education	56(29.1)	65(291)	0.78(0.453–1.372)	0.89(0.505–1.590)	0.7
	Secondary education	61(31.4)	45(20.2)	0.5(0.282–0.888)	0.59(0.332–1.07)	0.08
	Tertiary education	41(21.1)	60(26.9)	0.99(0.556–1.776)	1(0.563–1.83)	0.96
Occupation	Merchant	37(19.1)	45(20.2)	1		
	Housewife	48(19.1)	52(23.3)	1.2(0.69–2.11)		
	Government employee	69(14.9)	57(12.6)	0.8(0.40–1.56)		
	Daily laborer	25(12.9)	35(15.7)	0.94(0.50–1.79)		
	Others	33(17)	25(11.2)	1.15(0.58–2.25)		
Family history of schizophrenia	Yes	31(16)	6(2.7)	1	1	
	No	163(84)	217(97.3)	6.8(2.80–16.87)	6.3(2.55–15.77)	0.00**
Ever heard about schizophrenia	Yes	117(60.3)	125(56.1)	1		
	No	77(39.7)	98(43.9)	1.2(0.806–1.761)		

## Discussion

This study focuses on awareness and attitudes toward Schizophrenia by using a mixed study method among community members in Hossana Town. As a result, nearly 46.5% of individuals had a positive attitude toward patients with schizophrenia. This finding is lower than the study findings from Arba Minch Zuria District (63.2%) [20]. The possible reason for this discrepancy might be due to the difference in time of the studies conducted, the awareness level of the community, methodological differences, and the way of people lives. But it is higher than a study done among Residents of Hawassa City, Southern Ethiopia (37.5%) [24]. The possible reason might be a difference in the study setting as the current study was conducted in an urban. The urban residents have relatively better education, economy, and access to information compared to rural communities about mental care and positive attitude towards them.

This study reveals that eccentric behavior and wandering were the most frequently responded manifestations of schizophrenia by respondents. This finding was in line with the study from Arba Minch Zuria District [20] and the Zaire community [13] reported as one has to exhibit behavior that draws public attention and disturbs society, recognized as having a mental disorder. In the current study, participants reveal that substance misuse, and conflict with family as the common causes of schizophrenia. This finding concurred with the finding from Arba Minch Zuria District [20], Pakistan [12], and Nigeria [25]. It a matter that the identified situations exposed an individual to a stressful situation and thus increased the probability of developing schizophrenia in the long run. The finding from the focus group discussion is concurrent with this report as most of the time people with mental illness were a previous history of substance misuse. Thus there is a need for the community member should keep their environment free from substance misuse and exercise peaceful handling of the conflicts that may happen within the family. In addition to that, it gives the clue on how to provide more knowledge and increased awareness of the causes of mental illness and dispel the myths around the predisposing factors for the disorders. But the study was done on qualitative study participants in southern Ethiopia [24], Feresbet district residents [26], and Ghana [27] who respond to those traditional reasons as causes of schizophrenia. This difference might be explained by the social, cultural, and religious differences of the study participants.

The evidence from the current study shows us spirituality/tradition was the leading place for treatment as the respondents' preferable place of the treatment of schizophrenia that accounting for around eighty-eight percent followed by. This finding is in line with the studies of Hawassa city of Ethiopia [24], Dessie town, northeast Ethiopia [28] and Nigeria [29] spiritual/traditional treatment was identified as the most preferred place for the treatment of schizophrenia. But a study done in Tanzania Dodoma contradicts this finding as the majority of the respondents mentioned the best model of treatment as the hospital concurs [30]. The qualitative finding revealed by the focus group discussion participant supports the study as most patients with mental illness were better taken to spiritual/traditional places for treatment. Because I know one mental patient was going to a spiritual place and know he is better than from the previous condition. Thus, the need for awareness sessions for the community on possible treatment options for better effectiveness of modern antipsychotic therapies. It is essential because the majority of people in this study area seek treatment from spiritual/ traditional methods as a preferred method.

In this study majority of the participants disagree that genetics is the primary factor in the development of schizophrenia. This finding is in line with the study conducted in the Community based study in Nigeria [29] and Dodoma, Tanzania [30] where the majority of the participants disagree that genetics is the primary factor in the development of schizophrenia. The qualitative finding supports here that schizophrenia is not genetic but they believe it might be happening due to a curse. I did not agree with idea that schizophrenia is a genetic disease since it comes due to an evil eye or curse. Also, other FGD participants said that schizophrenia cannot happen or be transmitted by family, it is not related to family history because I know one patient with schizophrenia in my village but still, no one from that comes to be schizophrenia.

In this study, those respondents with a family history of schizophrenia were six times more likely to develop a positive attitude than those participants without a family history of schizophrenia. It might reveal that when there is the exposure of community members to patients with mental illness in the family they may adapt to the environment to handle the patients and tolerate the condition. And they believe that those patients may have the probability to get healthy when they get modern treatment. This study has the strength of addressing both quantitative and qualitative views to explore the factors in a different dimension. But it has some limitations in focusing only on attitude variables.

## Conclusion

In this study, the findings of this study indicate more than half of the participants had a negative attitude toward people with schizophrenia.

Eccentric behavior and wandering were the most commonly mentioned manifestation of schizophrenia. Substance misuse, loss of loved ones, and conflict with family have been perceived causes of schizophrenia in this study. Spiritual/traditional places were preferred places for the treatment of schizophrenia. Having a family history of schizophrenia was the only factor associated with attitude towards schizophrenia. Therefore, due attention should be given to changing negative attitudes towards schizophrenia, reducing potential schizophrenia-predisposing factors, and enhancing community awareness to seek medical help as early as possible when such kinds of events occur.

## Data Availability

The datasets of this study are available upon reasonable request from the corresponding author.
